# Supplementary material for: The bHLH-zip transcription factor SREBP regulates triterpenoid and lipid metabolisms in the medicinal fungus Ganoderma lingzhi
Source: Commun Biol. 2023 Jan 3;6:1. doi: 10.1038/s42003-022-04154-6 (PMC9810662; doi:10.1038/s42003-022-04154-6)

Supplementary Table 1 Gene annotation

Item	Percentage
All	100%
Annotation	97.54%
Uniprot	49.25%
Pfam	69.84%
Refseq	35.31%
NCBI nr	97.23%
GO	48.81%
KEGG	36.07%
Pathway	22.08%
COG	9.71%

Supplementary Table 2 Genes involved in terpenoid backbone and steroid biosynthesis in *G. lingzhi*

Gene ID	Functional annotation	Abbr.	KEGG
**Terpenoid backbone biosynthesis**			
g8260.t1	acetyl-CoA acetyltransferase	ACAT	K00626
g1941.t1	3-hydroxy-3-methylglutaryl CoA synthetase	HMGCS	K01641
g2396.t1	3-hydroxy-3-methylglutaryl CoA reductase	HMGCR	K00021
g3941.t1	mevalonate kinase	MK	K00869
g10716.t1	phosphomevalonate kinase	PMK	K00938
g6933.t1	di-phosphomevalonate decarboxylase	MVD	K01597
g640.t1	isopentenyl-diphosphate delta-isomerase	IDI	K01823
g3512.t1	farnesyl diphosphate synthase	FDPS	K00787
g11591.t1	farnesyl diphosphate synthase	FDPS	K00787
g1847.t1	squalene synthase	SQS	K00801
g10485.t1	squalene monooxygenase	SE/ERG1	K00511
g3089.t1	squalene monooxygenase	SE/ERG1	K00511
g1881.t1	lanosterol synthase	LSS/ERG7	K01852
**Ergosterol biosynthesis**			
g10251.t1	lanosterol 14-demethylase	CYP51/ERG11	K05917
g3908.t1	lanosterol 14-demethylase	CYP51/ERG11	K05917
g8913.t1	Delta14-sterol reductase	ERG24	K00222
g3236.t1	methyl sterol monooxygenase	ERG25	K07750
g10931.t1	sterol-4alpha-carboxylate 3-dehydrogenase	ERG26	K07748
g10015.t1	keto steroid reductase	ERG27	K09827
g10004.t1	keto steroid reductase	ERG27	K09827
g9990.t1	keto steroid reductase	ERG27	K09827
g10009.t1	keto steroid reductase	ERG27	K09827
g10002.t1	keto steroid reductase	ERG27	K09827
g10017.t1	keto steroid reductase	ERG27	K09827
g9995.t1	keto steroid reductase	ERG27	K09827

g7576.t1	sterol 24-C-methyltransferase	ERG6	K00559
g7787.t1	sterol isomerase	ERG2	K09829
g3976.t1	Delta7-sterol 5-desaturase	ERG3	K00227
g288.t1	sterol 22-desaturase	ERG5	K09831
g1634.t1	Delta 24-sterol reductase	ERG4	K00223
**Other secondary metabolic biosynthesis**			
g5910.t1	polycis-polyprenyl diphosphate synthase [(2E,6E)-farnesyl diphosphate specific	DHDDS	K11778
g1018.t1	hexaprenyl-diphosphate synthase	hexPS	K05355
g7691.t1	farnesyltransferase type-1 subunit alpha	FNTA	K05955
g10332.t1	farnesyltransferase type-1 subunit beta	FNTB	K05954
g2861.t1	farnesylcysteine lyase	FCLY	K05906
g4496.t1	protein-S-isoprenylcysteine O-methyltransferase	ICMT/STE14	K00587
g7836.t1	prenyl protein peptidase	RCE1/FACE2	K08658
g3038.t1	endopeptidase	STE24	K06013
g10426.t1	endopeptidase	STE24	K06013
g541.t1	lysosomal acid lipase/cholesteryl ester hydrolase	LIPA	K01052
g1629.t1	sterol O-acyltransferase	SOAT	K00637

Supplementary Table 3 SREBP target genes belonging to the cytochrome P450 genes

No.	Gene ID	SREBP binding DNA loci: Start-End	Distance to TSS	Matched sequence	Fitted motif
**1**	g5000	ctg6:2419681-2419971	158	GAGGAAGGAGA	GRVGRVGRVGR
**2**	g4875	ctg6:1984859-1985181	100	GGAGACGGGAA	GRVGRVGRVGR
**3**	g4164	ctg5:2505140-2505393	-1973	GAAGATGAGGA	GRVGRVGRVGR
**4**	g11088	ctg25:40482-40765	-40	GACAACGAGGA	GRVGRVGRVGR
**5**	g11110	ctg25:137809-138607	-1501	GAAAACGGCGA	GRVGRVGRVGR
**6**	g6752	ctg9:2105773-2106135	-8	TAAGACGA	GAMGACGR
**7**	g87	ctg1:427240-427483	160	GAGGAGGACGA	GRVGRVGRVGR
**8**	g10251*	ctg42:708348-708705	43	GAAGAAGGGGA	GRVGRVGRVGR
**9**	g3908*	ctg5:1519269-1519598	-107	GAGAACGAAGG	GRVGRVGRVGR
**10**	g7787*	ctg12:1080202-1080480	-99	GGAGAGGACGA	GRVGRVGRVGR

*The gene is involved in ergosterol biosynthesis and details showed in Supplementary Table 2.

Supplementary Figure 1 Estimation of *G. lingzhi* genome size by K-mer.

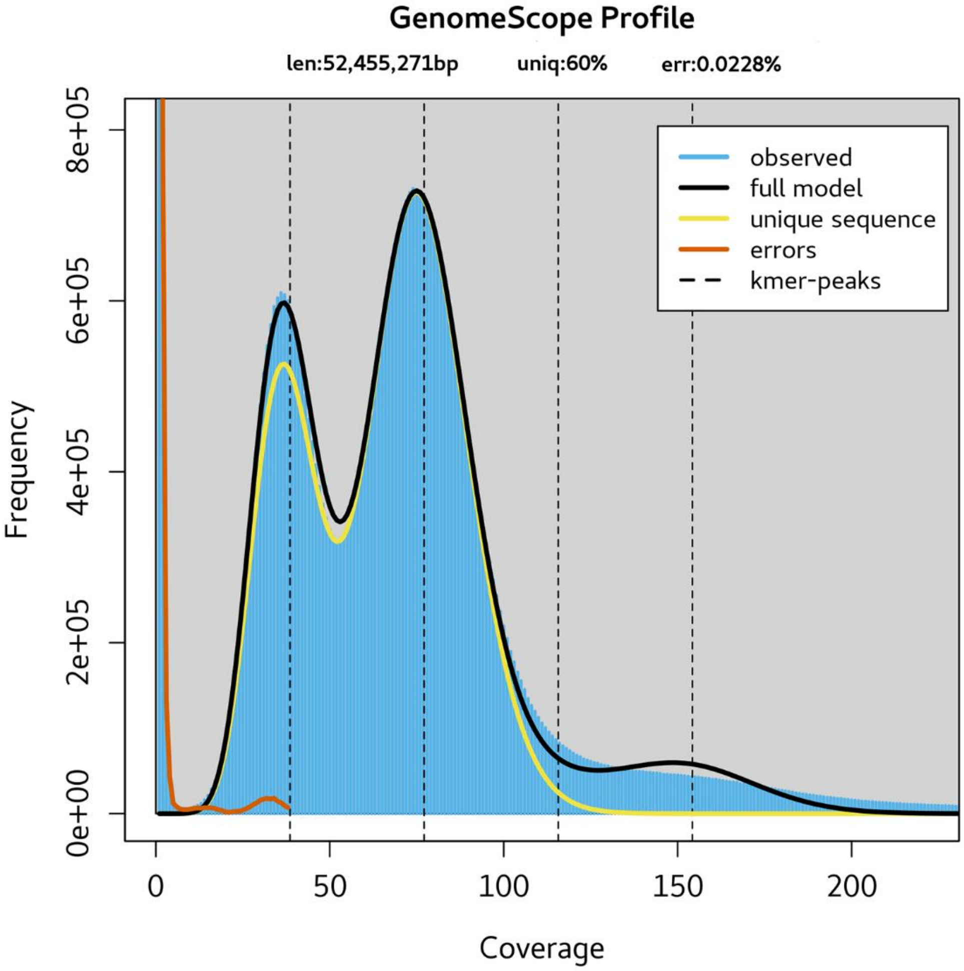

The blue line indicated the actual K-mer curve, the black line indicated the K-me curve estimated by the model, the yellow line indicated the K-mer curve corresponding to unique data, the red line K-mer the error curve caused by sequencing error, and the dotted line indicated the predicted K-mer peak. Estimated genome haploid length: 52,455,271 bp, unique length: 31,489,916 bp, heterozygosity: 1.505%, read error rate: 0.023%.

Supplementary Figure 2 Gene loci and sequence alignment of SREBP.

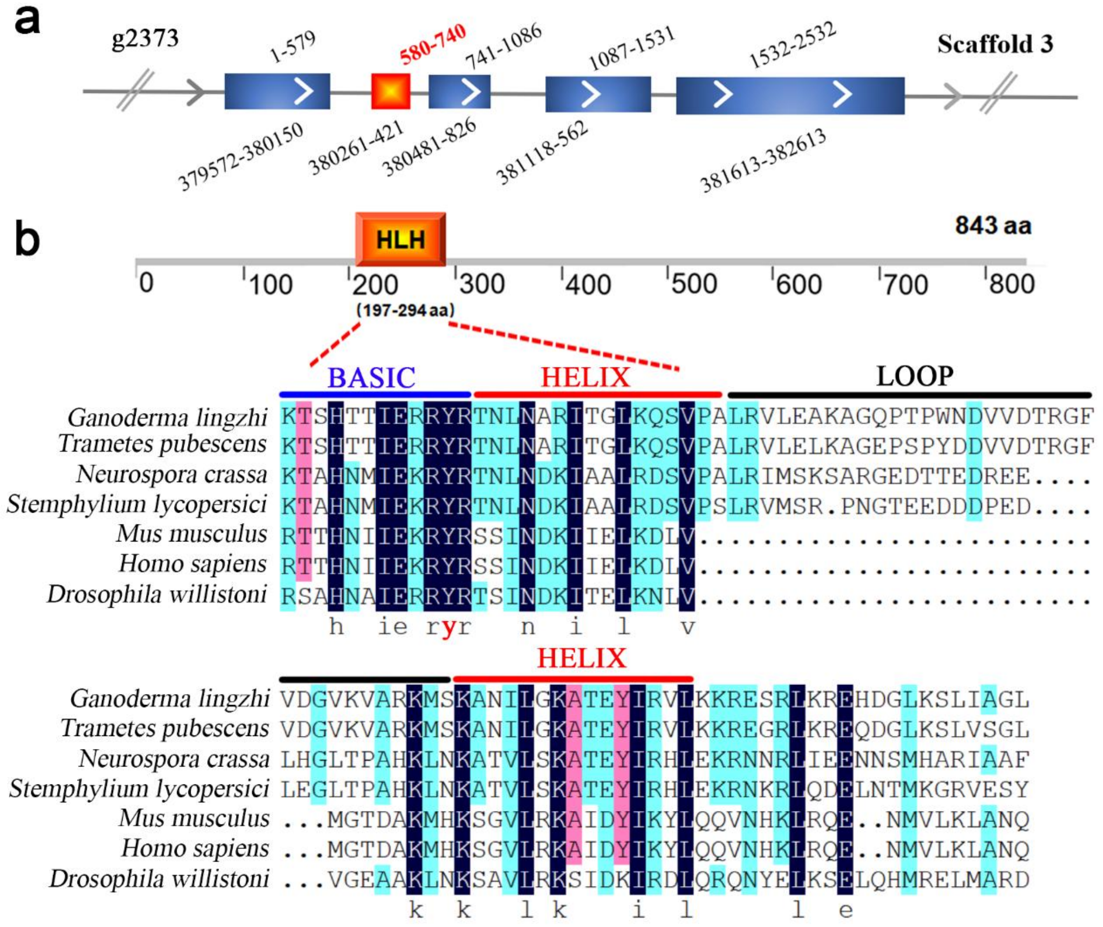

a The gene structure of the chromosomal regions containing the SREBP gene of *G. lingzhi*. The gene sequence encoding the bHLH domain is marked in red. **b** Conserve domain analysis of putative SREBP protein was calculated by NCBI Conserved Domains Database (http://www.ncbi.nlm.nih.gov/cdd). The bHLH domain sequence alignment was performed using DNAMAN software. Accession numbers for the aligned proteins: *Trametes pubescens*, OJT12715.1; *Neurospora crassa*, XP_960366.2; *Stemphylium lycopersici*, RAR12235.1; *Mus musculus*, NP_150087.1; Homo sapiens, Q12772.2; *Drosophila willistoni*, EDW73338.1.

Supplementary Figure 3 Four DNA motifs preferred by the *G. lingzhi* SREBPs.

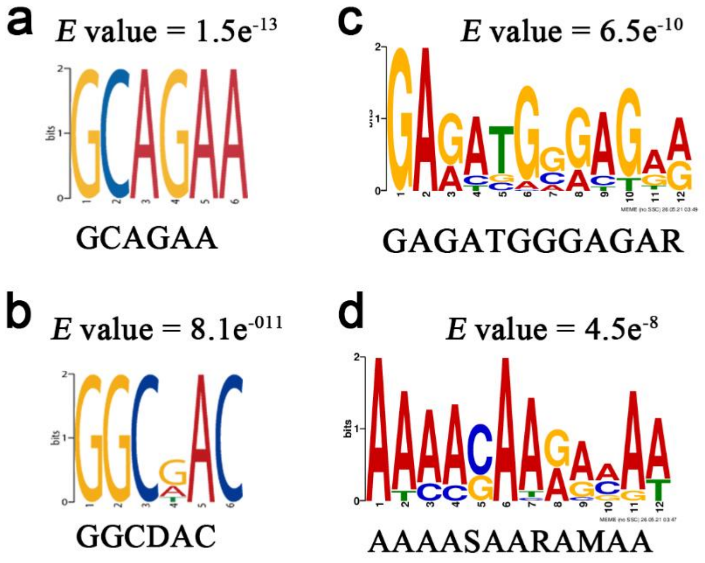

Shown are representative motifs derived from data sets Supplementary Date 4.

Supplementary Figure 4 The construction and characterization of OE::SREBP strains.

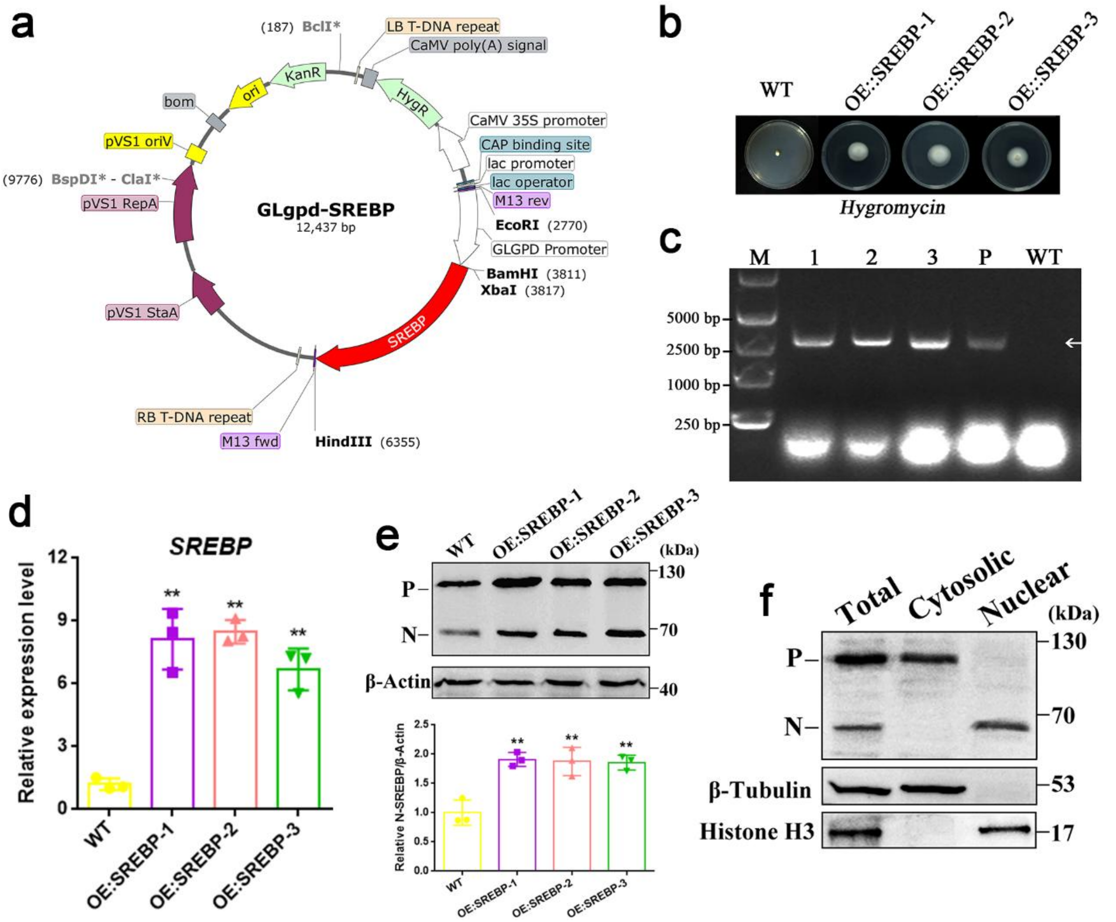

a Construction of SREBP overexpression plasmid. In the plasmid, transcription of hygromycin resistance gene and the target genes is driven by the 35S and gpd promoter, respectively. **b** Growth of WT strain and positive transformants in plates containing hygromycin. **c** The amplification pattern obtained with primers for the gpd promoter-SREBP fusion fragment (indicated by arrow) using genomic DNA isolated from the *G. lingzhi* transformants and WT. Lane P: plasmid as a positive control; Lane WT: WT strain as a negative control. **d** The relative mRNA levels of SREBP in *G. lingzhi* transformants and WT on shaking for 7 days in liquid cultures. The expression level of the SREBP gene in the WT strain was arbitrarily set to 1. The mean and standard error were determined using data from three independent replicates (n = 3, ***P* < 0.01 by two-way ANOVA). **e** Western blot analysis of the protein levels of SREBP in WT and OE::SREBP strains. P and N denote the full-length, precursor

SREBP and the cleaved, active SREBP, respectively. The histogram shows the N-SREBP/β-Actin ratio in the WT and OE::SREBP strains, and these values were obtained by integrating the signals from three independent experiments. The N-SREBP/β-Actin ratio in the WT strain was set to 1. The mean and standard error were determined using data from three independent replicates (n = 3, $^{**}P < 0.01$ by two-way ANOVA). **f** Western blot analysis of the total extract, cytosolic and nuclear fractions from the WT strain was performed to confirm the cytosolic and nuclear versions of SREBP protein.

Supplementary Figure 5 Summary of transcriptome sequencing data.

a

Sequencing data Statistics					
**Samples**	**Clean reads**	**Clean bases**	**GC Content**	**%≥Q30**	**Mapping ratio**
WT-1	20,104,482	6,008,812,788	58.36%	94.23%	92.84%
WT-2	24,084,022	7,201,952,206	59.50%	94.40%	92.43%
WT-3	22,693,494	6,787,606,572	59.46%	94.36%	92.48%
OE:SREBP-1	28,373,239	8,481,752,248	59.59%	94.43%	91.59%
OE:SREBP-2	22,765,670	6,797,022,502	59.56%	94.63%	91.64%
OE:SREBP-3	21,175,699	6,329,749,024	59.57%	94.57%	91.72%

b

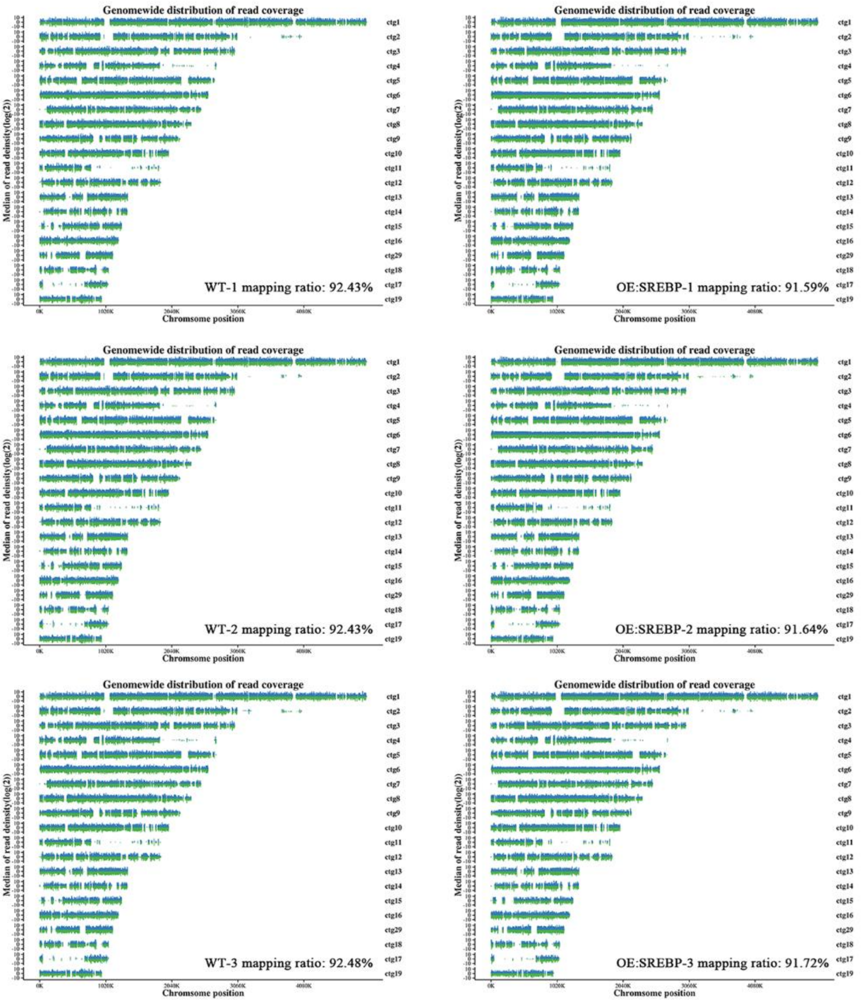

a The statistics of RNA sequencing data.≥ Q30%: Percentage of bases with Q-score no less than Q30. **b** Distribution of mapped reads on reference genome: position and depth. X-axis: Position on chromosome; Y-axis: Log2 of coverage depth (coverage depth was defined as reads counted within a chromosome window of 10 kb in length); Blue represents + strand and green represents – strand.

Supplementary Figure 6 Uncropped Western blots.

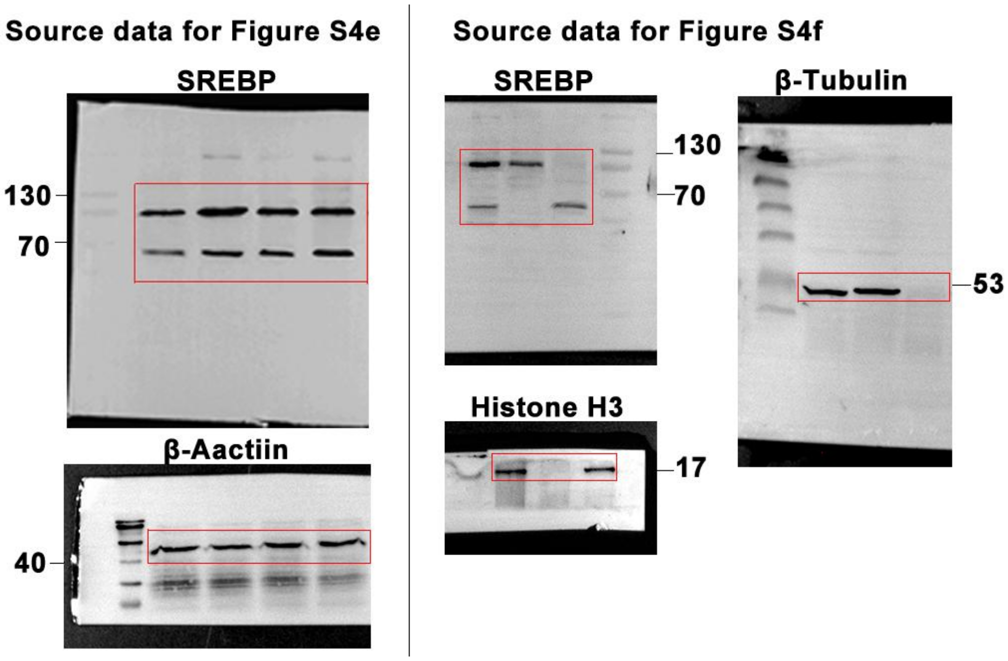

Supplement: Supplementary file 2 — Supplementary Information [file 42003_2022_4154_MOESM2_ESM.pdf]
