# Supplementary material for: The bHLH-zip transcription factor SREBP regulates triterpenoid and lipid metabolisms in the medicinal fungus Ganoderma lingzhi
Source: Commun Biol. 2023 Jan 3;6:1. doi: 10.1038/s42003-022-04154-6 (PMC9810662; doi:10.1038/s42003-022-04154-6)
Supplement: Supplementary file 3 — Description of Additional Supplementary Files [file 42003_2022_4154_MOESM3_ESM.pdf]

Description of Additional Supplementary Files

File name: Supplementary Data 1

Description: Gene annotation of G.lingzhi

File name: Supplementary Data 2

Description: Cytochrome P450

File name: Supplementary Data 3

Description: SREBP binding sites

File name: Supplementary Data 4

Description: SREBP binding motif

File name: Supplementary Data 5

Description: KEGG for SREBP targets

File name: Supplementary Data 6

Description: Novel transcripts annotation

File name: Supplementary Data 7

Description: Differentially expressed genes in RNA-seq

File name: Supplementary Data 8

Description: KEGG for UP-DEGs

File name: Supplementary Data 9

Description: Metabolic differences between OE::SREBP and WT strains

File name: Supplementary Data 10

Description: Oligonucleotides and primers

File name: Supplementary Data 11

Description: MS-MS Date of Gas

File name: Supplementary Data 12

Description: The annotated gff3 file

File name: Supplementary Data 13

Description: Raw data for Fig. 4e, 5d, S4d, S4e
