# Supplementary material for: The bHLH-zip transcription factor SREBP regulates triterpenoid and lipid metabolisms in the medicinal fungus Ganoderma lingzhi
Source: Commun Biol. 2023 Jan 3;6:1. doi: 10.1038/s42003-022-04154-6 (PMC9810662; doi:10.1038/s42003-022-04154-6)
Supplement: Supplementary file 13 — Supplementary Data 10 [file 42003_2022_4154_MOESM13_ESM.docx]

**Oligonucleotides and PCR primers used (5ʹ→3ʹ).**

| **For gel mobility shift assay** | | |
| --- | --- | --- |
| g3941*_pro_* | Forward | TGGGGCAGCCTGGACAGCGAAAAGCATGGATGGGA |
|  | Reverse | TCCCATCCATGCTTTTCGCTGTCCAGGCTGCCCCA |
| g1941*_pro_* | Forward | CACATCCCGCCCTCCTCTTCGTCCACGCCAGC |
|  | Reverse | GCTGGCGTGGACGAAGAGGAGGGCGGGATGTG |
| g4989*_pro_* | Forward | TCTCCGCTTCTCTTCTTCCTCTGAACCTT |
|  | Reverse | AAGGTTCAGAGGAAGAAGAGAAGCGGAGA |
| g3847*_pro_* | Forward | AGTGATAGGAGGAGGAGAGACTGGGGGACGGGGGTGCGAA |
|  | Reverse | TTCGCACCCCCGTCCCCCAGTCTCTCCTCCTCCTATCACT |
| g805*_pro_* | Forward | ATCCGCGAGAGCAGAGAGGGCGGAGGACGGGGTGGTG |
|  | Reverse | CACCACCCCGTCCTCCGCCCTCTCTGCTCTCGCGGAT |
| g4280*_pro_* | Forward | AGGGACACATGAGAAGGAGACGATTGAGACGGGAA |
|  | Reverse | TTCCCGTCTCAATCGTCTCCTTCTCATGTGTCCCT |
| g4309*_pro_* | Forward | ACGAGGTGTGAGCGAGGAGTGGGGAACTGC |
|  | Reverse | GCAGTTCCCCACTCCTCGCTCACACCTCGT |
| g1208*_pro_* | Forward | TTCACCCCGTTCGCCCCGTTCGCCCCGTTCGCCCCATTCACCCCGTTC |
|  | Reverse | GAACGGGGTGAATGGGGCGAACGGGGCGAACGGGGCGAACGGGGTGAA |
| g5041*_pro_* | Forward | GCTTGCTATTTTCGCCCTCTTCACCATCGACTT |
|  | Reverse | AAGTCGATGGTGAAGAGGGCGAAAATAGCAAGC |
| g6347*_pro_* | Forward | CTGGCAATCTTCTGGCATCTCTGATTCTGA |
|  | Reverse | TCAGAATCAGAGATGCCAGAAGATTGCCAG |
| **For quantitative RT-PCR** | | |
| g1914 | Forward | TCCATCAGCCCAAACCT |
|  | Reverse | ATTGGATGAAACGATACGC |
| g3941 | Forward | CTTCGCTGTTACGGTCTTAC |
|  | Reverse | GATCGCTGCCTTTGGGT |
| g7787 | Forward | CCGTTGTTTATGTTGCGTAG |
|  | Reverse | TCAGCGGTGAGGTTCGA |
| g3908 | Forward | ATCACTTGCCAGCCTACC |
|  | Reverse | TACGCCCGAACTTTCAT |
| g4309 | Forward | GCGGAGACTATGAAACCA |
|  | Reverse | AGGACGGCGATGAGAAG |
| g1208 | Forward | TAGATTACCCTCAGTGGATTG |
|  | Reverse | TGTCCCGACAGAAGAAAA |
| g4280 | Forward | GCGATCACGACTGGATG |
|  | Reverse | AGAGCCGAAATAGCTTACC |
| g4387 | Forward | CCGCCGTCTTGAACCCT |
|  | Reverse | CTCCGAGCCTCAGCATT |
| g4875 | Forward | ACAGCCTCCTTTCTTCCG |
|  | Reverse | ATGCGAGGATTTGTGCC |
| g4146 | Forward | GTGTTGGCGAAGTAGACG |
|  | Reverse | TTGTGAATTGGAGGCATT |
| g2373 | Forward | TGGCACTGTCGGAAACAC |
|  | Reverse | GCTCGGTCGCCTTAGAAC |
| *18S* | Forward | TCGAGTTCTGACTGGGTTGT |
|  | Reverse | TCCGTTGCTGAAAGTTGTAT |
| **For cloning** | | |
| SREBP | Forward | ATCG*tctaga*ATGTCCTCATCTTCCTCCG |
|  | Reverse | ATCG*aagctt*CTAGTCAAACGCCCTCCCG |
| bHLH domain | Forward | CGTCCGAAGACGAGCCA |
|  | Reverse | CCACTCCCTTTCCCACTCC |
| gpd-SREBP fusion | Forward | CTTGACGGTTCACTGGTTT |
|  | Reverse | CGCTCTTGCTCCTCCTT |
