# Supplementary material for: The bHLH-zip transcription factor SREBP regulates triterpenoid and lipid metabolisms in the medicinal fungus Ganoderma lingzhi
Source: Commun Biol. 2023 Jan 3;6:1. doi: 10.1038/s42003-022-04154-6 (PMC9810662; doi:10.1038/s42003-022-04154-6)
Supplement: Supplementary file 14 — Supplementary Data 11 [file 42003_2022_4154_MOESM14_ESM.docx]

| **Index** | **Compounds** | **m/z** | **Fragment Ions** |
| --- | --- | --- | --- |
| kz002245 | Ganoderic Acid SZ | 453.3 | 453.3,435.3,185.1,187.1,239.2,173.1,201.2,225.2 |
| kz002248 | Ganoderic Acid F | 455.4 | 455.4,437.3,455.4,229.2,299.3,123.1,201.2,135.1 |
| kz002252 | Ganoderic Acid Jb | 471.3 | 471.3,435.3,453.3,201.2,187.1,471.3,159.1,175.1 |
| kz002255 | Ganodermanontriol | 473.4 | 473.4,329.2,455.4,415.3,243.2,261.2,437.3,189.1 |
| kz002270 | Ganoderic Acid T-Q | 511.3 | 511.3,433.3,493.3,311.2,451.3,293.2,399.3,337.2 |
| kz002273 | Ganoderic Acid Mf | 513.4 | 513.4,435.3,495.3,201.2,295.2,203.2,453.3,133.1 |
| kz002278 | Ganoderenic Acid C | 517.3 | 517.3,371.3,499.3,399.3,463.3,481.3,353.2,381.2 |
| kz002279 | Ganoderic Acid C2 | 519.3 | 519.3,355.3,519.3,483.3,501.3,465.3,447.3,373.3 |
| kz002283 | Ganoderic Acid V | 529.4 | 529.4,469.3,243.2,423.3,329.2,451.3,369.3,355.3 |
| kz002288 | Ganoderic Acid Mj | 545.2 | 545.2,527.3,449.3,467.3,431.3,353.2,421.3,327.2 |
| kz002289 | Ganoderic Acid R | 555.4 | 555.4,435.3,495.3,201.2,187.1,145.1,239.2,341.2 |
| kz002290 | Ganoderic Acid Me | 555.4 | 555.4,435.3,495.3,295.2,201.2,203.2,187.1,189.2 |
| kz002291 | Ganoderic Acid Mk | 571.4 | 571.4,433.3,451.3,493.3,201.2,511.3,293.2,339.2 |
| kz002293 | Ganoderic Acid AP2 | 587.4 | 587.4,569.4,491.3,509.3,431.3,395.3,409.3,463.3 |
| kz002294 | Ganoderic Acid T | 613.4 | 613.4,433.3,493.3,553.4,201.2,293.2,451.3,227.2 |
| LZGA0029 | Deacetyl Ganoderic acid F | 527.3 | 527.4,509.6,479.4,465.5,435.6,365.8,315.2,299.4 |
| LZGA0042 | Ganoderic acid A | 515.3 | 515.5,497.5,453.6,435.5,355.3,337.3,299.5,285.5 |
| LZGA0071 | Ganoderic acid GS-3 | 557.3 | 539.6,497.4,453.5,435.6,303.4,287.4 |
| LZGA0109 | Ganoderic acid W | 571.4 | 553.6,523.8,509.3,479.3,465.4,419.6,345.5,303.5,285.4 |
| lztp000125 | Ganochlearic acid A | 401.2 | 357.4,329.4,313.3,287.4 |

kz002245

kz002248

kz002252

kz002255

kz002270

kz002273

kz002278

kz002279

kz002283

kz002288

kz002289

kz002290

kz002291

kz002293

kz002294

LZGA0029

LZGA0042

LZGA0071

LZGA0109

lztp000125
