# Supplementary material for: The bHLH-zip transcription factor SREBP regulates triterpenoid and lipid metabolisms in the medicinal fungus Ganoderma lingzhi
Source: Commun Biol. 2023 Jan 3;6:1. doi: 10.1038/s42003-022-04154-6 (PMC9810662; doi:10.1038/s42003-022-04154-6)
Supplement: Supplementary file 17 — Reporting Summary [file 42003_2022_4154_MOESM17_ESM.pdf]

nature portfolio

Corresponding author(s): DBPR COMMSBIO-22-2482B

Last updated by author(s): Oct 12, 2022

Reporting Summary

Nature Portfolio wishes to improve the reproducibility of the work that we publish. This form provides structure for consistency and transparency in reporting. For further information on Nature Portfolio policies, see our Editorial Policies and the Editorial Policy Checklist.

Statistics

For all statistical analyses, confirm that the following items are present in the figure legend, table legend, main text, or Methods section.

n/a	Confirmed	
☐	☒	The exact sample size (*n*) for each experimental group/condition, given as a discrete number and unit of measurement
☐	☒	A statement on whether measurements were taken from distinct samples or whether the same sample was measured repeatedly
☐	☒	The statistical test(s) used AND whether they are one- or two-sided<br>*Only common tests should be described solely by name; describe more complex techniques in the Methods section.*
☐	☒	A description of all covariates tested
☐	☒	A description of any assumptions or corrections, such as tests of normality and adjustment for multiple comparisons
☐	☒	A full description of the statistical parameters including central tendency (e.g. means) or other basic estimates (e.g. regression coefficient) AND variation (e.g. standard deviation) or associated estimates of uncertainty (e.g. confidence intervals)
☐	☒	For null hypothesis testing, the test statistic (e.g. F, t, r) with confidence intervals, effect sizes, degrees of freedom and P value noted<br>*Give P values as exact values whenever suitable.*
☐	☒	For Bayesian analysis, information on the choice of priors and Markov chain Monte Carlo settings
☐	☒	For hierarchical and complex designs, identification of the appropriate level for tests and full reporting of outcomes
☐	☒	Estimates of effect sizes (e.g. Cohen's *d*, Pearson's *r*), indicating how they were calculated

Our web collection on statistics for biologists contains articles on many of the points above.

Software and code

Policy information about availability of computer code

Data collection	No unreported custom computer code or algorithm was used.
Data analysis	No unreported custom computer code or algorithm was used.

For manuscripts utilizing custom algorithms or software that are central to the research but not yet described in published literature, software must be made available to editors and reviewers. We strongly encourage code deposition in a community repository (e.g. GitHub). See the Nature Portfolio guidelines for submitting code & software for further information.

Data

Policy information about availability of data

All manuscripts must include a data availability statement. This statement should provide the following information, where applicable:

- Accession codes, unique identifiers, or web links for publicly available datasets
- A description of any restrictions on data availability
- For clinical datasets or third party data, please ensure that the statement adheres to our policy

The genome assembly reported in this paper is associated with NCBI BioProject: PRJNA738334 and BioSample: SAMN19718602. The detailed sequencing information have been deposited in NCBI's Sequence Read Archive under accession numbers SRR14933280 and SRR14933281. An annotated gff3 file was shown in Supplementary Data 12. DAP-seq and RNA seq data underlying the findings described in the manuscript are fully available without restriction from the Bioproject

Sequence Read Archive: SRR21374162-64 and SRR20305604-09, respectively. The raw data for Fig. 4e, 5d, S4d, S4e were shown in Supplementary Data 13. Uncropped western blots are in Supplementary Figure 6.

Human research participants

Policy information about studies involving human research participants and Sex and Gender in Research.

Reporting on sex and gender	This study don't involve human research participants and Sex and Gender in Research.
Population characteristics	This study don't involve human research participants and Sex and Gender in Research.
Recruitment	This study don't involve human research participants and Sex and Gender in Research.
Ethics oversight	This study don't involve human research participants and Sex and Gender in Research.

Note that full information on the approval of the study protocol must also be provided in the manuscript.

Field-specific reporting

Please select the one below that is the best fit for your research. If you are not sure, read the appropriate sections before making your selection.

☒ Life sciences ☐ Behavioural & social sciences ☐ Ecological, evolutionary & environmental sciences

For a reference copy of the document with all sections, see nature.com/documents/nr-reporting-summary-flat.pdf

Life sciences study design

All studies must disclose on these points even when the disclosure is negative.

Sample size	No sample-size calculation was performed.
Data exclusions	No date were excluded from the analyses.
Replication	The data from three independent replicates.
Randomization	Our research sample is fungus Ganoderma lingzhi, don't involve randomization.
Blinding	This study don't involve blinding.

Reporting for specific materials, systems and methods

We require information from authors about some types of materials, experimental systems and methods used in many studies. Here, indicate whether each material, system or method listed is relevant to your study. If you are not sure if a list item applies to your research, read the appropriate section before selecting a response.

Materials & experimental systems

n/a	Involved in the study	
☐	☒	Antibodies
☒	☐	Eukaryotic cell lines
☒	☐	Palaeontology and archaeology
☒	☐	Animals and other organisms
☒	☐	Clinical data
☒	☐	Dual use research of concern

Methods

n/a	Involved in the study	
☒	☐	ChIP-seq
☒	☐	Flow cytometry
☒	☐	MRI-based neuroimaging

Antibodies

Antibodies used	β-Actin antibody; β-Tubulin antibody; Histone-H3 antibody; polyclonal antibody against SREBP.
Validation	β-Actin antibody (1:2000, AT0097, CMCTAG); β-Tubulin antibody (1:2000, AT0003, CMCTAG); Histone-H3 antibody (1:2000, AT0005, CMCTAG) ;polyclonal antibody against SREBP (immunization of rabbits with the SREBP-bHLH protein by a professionally qualified antibody preparation company). The results of Western blot analysis using these antibodies are shown in Figure S4.
